# Iridoid from *Eucommia ulmoides* Oliv. Exerts Antiarthritis Effects by Inhibiting the JAK2/STAT3 Signaling Pathway *In Vivo* and *In Vitro*

**DOI:** 10.1155/2023/4167906

**Published:** 2023-04-19

**Authors:** Li-Dong Tang, Jian-Ying Wang, Yan Zhang, Xiao-Yun Chen, Lei Zhang, Ying Yuan

**Affiliations:** ^1^School of Traditional Chinese Materia Medica, Shanghai University of Traditional Chinese Medicine, No. 1200 Cailun Road, Pudong District, 201203 Shanghai, China; ^2^Shanghai Innovation Center of TCM Health Service, Shanghai University of Traditional Chinese Medicine, No. 1200 Cailun Road, Pudong District, Shanghai 201203, China; ^3^Shanghai Longhua Hospital Affiliated to Shanghai University of TCM, Rheumatoid Department, No. 725 South Wanpin Road, Xuhui District, Shanghai 200232, China

## Abstract

The purpose of this study was to investigate the anti-inflammatory effects of EU-Idd both *in vivo* and *in vitro*. *In vivo,* we used the collagen-induced arthritis (CIA) rat model to investigate the efficacy of EU-Idd on rheumatoid arthritis. Hematoxylin-eosin staining and Safranin O-fast green staining were used to evaluate the pathological status of the ankle joints in CIA rats. Micro-CT scanning was used to investigate bone erosion of the ankle joints. *In vitro*, the effect of EU-Idd on Th17 cell differentiation was identified by flow cytometry. TRAP staining was used to detect osteoclast cells. HFLS-RA model cells, induced by tumor necrosis factor-*α*(TNF-*α*), were used to evaluate the anti-inflammatory effects of EU-Idd while the levels of related inflammatory cytokines and JAK2/STAT3 proteins were detected by RT-qPCR and western blotting. EU-Idd alleviated joint inflammation in CIA rats and exerted protective effects on the ankle joints. EU-Idd also prevented the differentiation of CD4^+^ T cells into Th17 cells, reduced the number of osteoclasts, and improved the expression levels of bone metabolism-related proteins including OPG and RANKL. Moreover, EU-Idd inhibited the invasion and migration of HFLS-RA cells and downregulated the expression of related inflammatory cytokine genes and the protein expression levels of p-JAK2 and p-STAT3, both *in vivo* and *in vitro*. EU-Idd exerts anti-inflammatory and osteoprotective effects by regulating the JAK2/STAT3 pathway in rheumatoid arthritis. These results are beneficial to excavate new pharmaceutical ingredients for rheumatoid arthritis from iridoid.

## 1. Introduction

Rheumatoid arthritis (RA) is an inflammatory autoimmune disease that is mainly characterized by inflammation of the synovium, joint destruction, and bone erosion, eventually leading to joint deformity and the loss of joint function [1]. Modern pharmacological studies have found that T helper 17 (Th17) cells are the predominant source of interleukin-17 (IL-17) production and are involved in the occurrence and development of various autoimmune diseases, chronic inflammatory diseases, and tumors [2]. During the development of RA, interleukin-23 (IL-23) interacts with its receptor to activate the downstream Janus kinase 2 (JAK2) signal transducer and activator of transcription (STAT) signaling pathway and simultaneously induces the differentiation of CD4^+^ T cells into Th17 cells [3, 4], thus increasing the levels of IL-17 cytokines [5]. In RA, IL-17 induces synovial fibroblasts and osteoblasts to activate the receptor activator of NF-*κ*B ligand (RANKL)/receptor activator of NF-*κ*B (RANK) signaling pathway associated with osteoclastogenesis, thus resulting in the destruction of bone [6, 7].

It has been found that both ancient and modern prescriptions containing *E. ulmoides* were mainly used to treat osteoarthritis [8]. Iridoid is isolated from the ethyl acetate fraction of *Eucommia ulmoides* Oliv. In our previous research, we showed that extracts from different parts of *E. ulmoides* could inhibit inflammation and bone destruction in a rat model of collagen-induced arthritis (CIA). For example, an ethyl acetate extract of *E. ulmoides* was found to reduce arthritis scores and downregulate the expression levels of p-65 and p-IKK proteins in CIA rats [9, 10]. The male flower of *E. ulmoides* has also been shown to exert antiarthritis effects by acting on the nuclear factor-*κ*B (NF-*κ*B) pathway [11]. A previous study indicated that the iridoids of *E. ulmoides* may promote the proliferation and differentiation of human osteoblasts and exert protective effects on bone [12]. However, the precise therapeutic effects of iridoids of *E. ulmoides* on RA are yet to be investigated. Therefore, in the present study, we comprehensively investigated the effects of EU-Idd on inflammation and bone metabolism in a rat model of CIA, with particular emphasis on Th17 cells and differentiation from RAW264.7 cells to osteoclasts and HFLS-RA cells.

## 2. Materials and Methods

### 2.1. Animals

A total of 32 female rats (120 ± 10 g) were obtained from Beijing CRL Laboratory Animal Co. Ltd. (Beijing, China). All animal protocols were approved by the Laboratory Animal Welfare and Animal Experimental Ethics Committee of Shanghai University of Traditional Chinese Medicine (approval number: PZSHUTCM200717004). Food and water were provided *ad libitum.*

### 2.2. CIA Induction and Treatment

**Figure 1:**
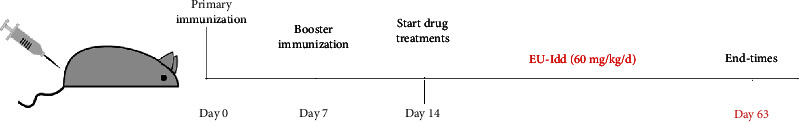
Flowchart depicting animal experimentation.

Figure 1 shows a flowchart depicting the animal experiments. In brief, a 2 mg/mL solution of bovine collagen II (CII) (Chondrex, Redmond, WA, USA) was prepared with glacial acetic acid; after refrigeration at 4°C overnight, the solution was mixed with an equal volume of complete Freund's adjuvant (CFA) (Chondrex, Redmond, WA, USA) to prepare a CII/CFA emulsion. Female rats were randomly assigned to four groups: a blank group (Blank, *n* = 8), a CIA model group (CIA, *n* = 8), a group treated with iridoids of *E. ulmoides* (EU-Idd, *n* = 8), and a group treated with *Tripterygium glycosides* (TG, *n* = 8). Iridoids of *E. ulmoides* were purchased from Daosifu Biotechnology Co. Ltd. (batch number: 180211A, Nanjing, China), High-performance liquid chromatography (HPLC) was used to quantify iridoids of *E. ulmoides*, and the results are shown in Figure S1. TG was purchased from Fudan Fuhua Pharmaceutical Co. Ltd. (Shanghai, China). Except for the Blank group, the other groups were injected with 0.1 mL of emulsion in the back and the tail. One week later, booster immunization was performed. After successful modeling, the rats in the Blank and CIA groups were intragastrically administered with distilled water while the EU-Idd and TG groups were given 60 mg/kg of EU-Idd and 5.4 mg/kg of TG, respectively, once a day for 7 weeks. The body weight, paw volume, and arthritis index (AI) of each rat were recorded every 7 days. AI was scored as follows: 0, no redness; 1, red spots or mild swelling of the ankle; 2, moderate swelling of the ankle; 3, severe swelling of the ankle; and 4, deformed ankle with walking difficulty. On day 63, the rats were anesthetized for specimen collection. We also used EU-Idd at a dose of 120 mg/kg, but the anti-inflammatory effect was not obvious (as shown in Figure S2). Therefore, we did not conduct follow-up inflammatory factor detection. After comprehensive literature survey results, we chose the concentration of 60 mg/kg as the final dose.

### 2.3. Histological Analysis of the Ankle Joints

For histological analysis, the left hind ankle joint of rats in each group was fixed in 10% neutral formaldehyde solution (China National Pharmaceutical Group Corporation, Beijing, China) and decalcified with EDTA Decalcified Solution (Sangon Biotech, Shanghai, China). Fixed joints were then paraffin-embedded and cut into sections (5 *μ*m thick). Hematoxylin-eosin (HE) staining was used to explore pannus formation and synovial hyperplasia in the ankle joints. Histological scores were calculated blindly using an established scoring system [13]: 0, normal joint structure, cartilage, and synovial tissue morphology; 1, a small amount of pannus formation, synovial hyperplasia, and no cartilage and bone destruction; 2, a large number of pannus had begun to form, a large amount of synovial hyperplasia, and slight cartilage destruction; 3, massive pannus formation, extensive synovial hyperplasia, and increased cartilage erosion; and 4, destruction of joint structure along with severe erosion of cartilage and bone. Safranin O-fast green (Solarbio, Beijing, China) staining was used to further investigate cartilage destruction; cartilage was scored using an established system [14]: 0, the cartilage structure showed no damage; 1, the cartilage surface was slightly damaged and appeared rough with cracks; 2, the cartilage was slightly to moderately damaged; 3, the local cartilage was severely damaged; and 4, a large area of the cartilage was damaged.

### 2.4. Micro-Computed Tomography (Micro-CT) of the Ankle Joint

To investigate bone erosion in the CIA rats, the right hind ankle joint was scanned using a high-resolution micro-CT system (SkyScan 1176, Bruker, Germany) after immersion in absolute ethanol. The scanning layer thickness was 18 *μ*m and system analysis software was used to analyze key microstructural parameters of the bone tissue in a region of interest, such as the bone volume/total volume fraction (BV/TV) and trabecular separation (Tb.Sp); the analysis range remained the same for all specimens.

### 2.5. Flow Cytometric Analysis

Peripheral blood monocytes and splenocytes were isolated from the peripheral blood and spleen. Cells were then resuspended in RPMI-1640 (Meilun Biotechnology, Dalian, China) supplemented with 10% FBS (Thermo Fisher Scientific, MA, USA) and 1% penicillin-streptomycin (Gibco, Grand Island, NY, USA) at a density of 1 × 10^6^/mL. The cells were then stimulated for 1 h with Cell Stimulation Cocktail (eBioscience, San Diego, CA, USA) following surface labeling with a FITC-CD4 mAb (Invitrogen, Carlsbad, CA, USA) and then cultured overnight in 5% CO_2_/95% air at 37°C. The next day, Cytofix/Cytoperm solution (BD, Franklin Lakes, NJ, USA) was added to the cells which were then stained with PE-IL-17 (Invitrogen, Carlsbad, CA, USA). The proportion of Th17 cells was quantitatively determined by flow cytometry (CytoFlex S, BD, Franklin Lakes, NJ, USA). Finally, results were analyzed by FlowJo software.

### 2.6. Cell Culture

CD4^+^ T cells were isolated from CIA rats and used to differentiate into Th17 cells. The Th17 cells were then cultured in RPMI-1640 medium supplemented with 10% FBS and 1% penicillin-streptomycin. RAW264.7 cells were purchased from Shanghai Cell Bank, Chinese Academy of Sciences (Shanghai, China). HFLS-RA cells were purchased from Huatuo Biotechnology Co. Ltd. (Guangzhou, China). The two cell types were cultured in DMEM (Meilun Biotechnology, Dalian, China) supplemented with 10% FBS and 1% penicillin-streptomycin. All cell cultures were incubated at 37°C and 5% CO_2_.

### 2.7. Isolation and Magnetic Enrichment of CD4^+^ T Cells

The spleens of rats in the Blank and CIA model groups were cut into pieces in a vertical flow clean bench and then lysed with red blood cell lysing solution (Biosharp, Hefei, China) to obtain splenocytes. Anti-CD4 was then added to the cells and incubated for 2 h. Next, we added sterile immunomagnetic magnetic beads and CD4+T cells were adsorbed by a magnetic rack. Finally, the purity of the CD4^+^ T cells was detected by flow cytometry.

### 2.8. Cell Viability Assays

Cell viability assays were carried out with a CCK-8 kit (Meilun Biotechnology, Dalian, China). In brief, Th17, RAW264.7, and HFLS-RA cells were seeded into a 96-well plate at a density of 1 × 10^5^/well and treated with EU-Idd at concentrations ranging from 10 to 640 *μ*g/mL for 24 h. Dimethyl sulfoxide (DMSO) was used to dissolve EU-Idd which was then diluted by 1000-fold in DMEM supplemented with 10% FBS and 1% penicillin-streptomycin. After culture, 10% CCK-8 medium was added to each well for 30 min. Results were detected at 450 nm with a microplate reader (BioTek Synergy 3); cell viability (%) = [OD (treated) − OD (blank)]/[OD (control) − OD (blank)] × 100.

### 2.9. The Induction of Th17 Differentiation

CD4^+^ T cells were isolated from rats in the Blank and CIA model groups. TGF-*β* (3 ng/mL), IL-6 (40 ng/mL), TNF-*α* (20 ng/mL), IL-1*β* (10 ng/mL), and IL-23 (30 ng/mL) were used to induce the differentiation of Th17 cells from CD4^+^ T cells. The cells were then seeded in 6-well plates with anti-CD3 (ab5690, Abcam, Cambridge, MA, USA) and anti-CD28 (ab243228, Abcam, Cambridge, MA, USA) and induced for 5 days; the medium was changed every other day. EU-Idd (20, 30, and 40 *μ*g/mL) treatment was given on the first day of induction. The proportion of Th17 cells was detected by flow cytometry after induction.

### 2.10. Griess Assays for RAW264.7 Cells

RAW264.7 cells were seeded into a 96-well plate at a density of 5 × 10^4^/well and pretreated with various concentrations of EU-Idd (0, 10, 20, 40, 80, 160, 320, and 640 *μ*g/mL) for 2 h. Then, the model and EU-Idd treatment groups were stimulated by LPS (Sigma, St. Louis, MO, USA) for 22 h. Griess medium was then added to the supernatant of cells for 10 min. When the reaction was complete, the OD value was read at 540 nm with a microplate reader. The relative rate of the NO release was expressed as a proportion (%) of the control group.

### 2.11. TRAP Assays for Osteoclasts

RAW264.7 cells were seeded into a 24-well plate at a density of 5 × 10^4^/well and incubated in osteogenic inducing medium composed of RANKL (100 ng/mL) and M-CSF (50 ng/mL) which was purchased from PeproTech (Rocky Hill, NJ, USA). The control group was treated with DMEM supplemented with 10% FBS and 1% penicillin-streptomycin while the model and EU-Idd (40, 80,160 *μ*g/mL) groups were induced for 7 days; the solution was changed every 2 days. After induction, the shape of the osteoclasts was clearly evident by microscopy; then, the osteoclasts were stained with a TRAP staining kit in accordance with the manufacturer's instructions (Sigma, St. Louis, MO, USA).

### 2.12. Migration and Invasion Assays for HFLS-RA Cells

Migration and invasion assays for HFLS-RA cells were performed using Transwell chambers and Ibidi inserts. HFLS-RA cells were seeded into a 24-well culture plate at a final concentration of 1 × 10^5^/mL. For migration assays, serum-free DMEM was added to the upper chambers while DMEM supplemented with 20% FBS and various concentrations of EU-Idd (40, 80, and 160 *μ*g/mL) was added to the lower well. Except for the negative group (containing only cells with DMEM supplemented with 20% FBS), both the model group and the treatment group were stimulated with TNF-*α* (PeproTech, Rocky Hill, NJ, USA) for 24 h; then, all cells were fixed by 4% paraformaldehyde and stained with 0.1% crystal violet. To assess the rate of migration, 33% acetic was used to elute the stained cells and the eluted solution was read at 570 nm. Migration rate (%) = [OD (treated) − OD (blank)]/[OD (control) − OD (blank)] × 100. For the invasion assays, Ibidi inserts were placed into a 24-well plate; similar experiments were applied to the cells. The scratched areas were photographed at 0 h and 24 h; healing rate (%) = [*S* (0 h) − *S* (24 h)]/*S* (0 h) × 100.

### 2.13. ELISA

The serum levels of TNF-*α*, IL-17, and IL-23 from CIA rats were measured by ELISA kits in accordance with the manufacturer's instructions. The same experimental method was also applied to detect the concentrations of IL-1*β* and IL-6 in the supernatant of HFLS-RA cells. The cells were treated with concentrations of 40, 80, and 160 *μ*g/mL and stimulated by TNF-*α* for 24 h. Then, the cells were centrifuged to obtain a cell supernatant for ELISA. Standard curves were calculated according to the OD value so that we could determine the concentrations of inflammatory cytokines. We purchased the following ELISA kits from Multi Sciences (Hangzhou, China): rat TNF-*α* ELISA Kit (70-EK382/3-96), rat IL-17A ELISA Kit (70-EK317/3-96), human IL-6 ELISA Kit (70-EK106/2-96), and human IL-1*β* ELISA Kit (70-EK101B-96). An IL-23 ELISA kit (E-EL-R0569c) was obtained from Elabscience (Wuhan, China).

### 2.14. RT-qPCR

Real-time quantitative PCR (RT-qPCR) was used to detect the levels of mRNA in spleen and cartilage tissue of rats and HFLS-RA cells stimulated by TNF-*α*. We used Tissue RNA Purification Kit PLUS (EZB-RN001-plus) and EZ-press RNA Purification Kit (B0004DP) to isolate total RNA *in vivo* and *in vitro*. To obtain cDNA, we used a Color Reverse Transcription Kit (A0010CGQ) to reverse transcribe RNA. The mRNA expression levels were determined by 2 × Color SYBR Green qPCR Master Mix (A0012-R2) on an ABI-7500 qPCR system (Thermo Fisher Scientific, USA) according to the manufacturer's instructions. The thermocycling conditions were as follows: 95°C for 5 min, 95°C for 10 s, 60°C for 30 s (40 cycles), and then 72°C for 90 s. The primer sequences are shown in Table 1. Data were normalized to *GAPDH* expression using the 2^−ΔΔCt^ method. The reagents used for RT-qPCR experiments were purchased from EZBioscience (Roseville, MN, USA).

### 2.15. Western Blotting

Total protein was extracted from spleen and cells by lysis buffer (Thermo Fisher Scientific, MA, USA) with 10% protease inhibitor and 10% phosphatase inhibitor (Roche, Basel, Switzerland). BCA kit (Meilun Biotechnology, Dalian, China) was used to determine the total protein concentration for each extract. Then, 40 *μ*g of protein from each sample was separated by 10% SDS-PAGE (EpiZyme, Shanghai, China) and electrophoretically transferred onto PVDF membranes (Millipore, Germany). The membranes were blocked with 3% non-fat milk (Sangon Biotech, Shanghai, China) eluted by tris-buffered saline with Tween 20 (TBST) and incubated with primary antibodies overnight at 4°C. The next morning, a HRP secondary antibody was incubated with the membranes for 1 h. Protein signals were then detected with an enhanced chemiluminescence kit (EpiZyme, Shanghai, China) according to the manufacturer's instructions. The intensity of each protein band was normalized to *β*-actin and GAPDH using ImageJ software. AK2 (#3230), p-JAK2 (#3776), STAT3 (#9139), p-STAT3 (#9145), RANK (#4845), TRAF6 (#67591), and anti-rabbit IgG (#7074) were all purchased from Cell Signaling Technology (Danvers, MA, USA). OPG (ab183910), RANKL (ab62516), and CTSK (ab187647) were all purchased from Abcam (Cambridge, MA, USA).

### 2.16. Statistical Analysis

SPSS 26.0 (IBM Corp., Armonk, NY, USA) was used for the statistical analysis of experimental data. Data are expressed as mean ± standard deviation. One-way ANOVA with Student's *t*-test and Dunnett's test was used for multiple comparisons. *P*  <  0.05 was considered significant.

## 3. Results

### 3.1. EU-Idd Inhibited Joint Inflammation and Bone Destruction in CIA Rats

#### 3.1.1. EU-Idd Alleviated Inflammatory Symptoms in CIA Rats

**Figure 2:**
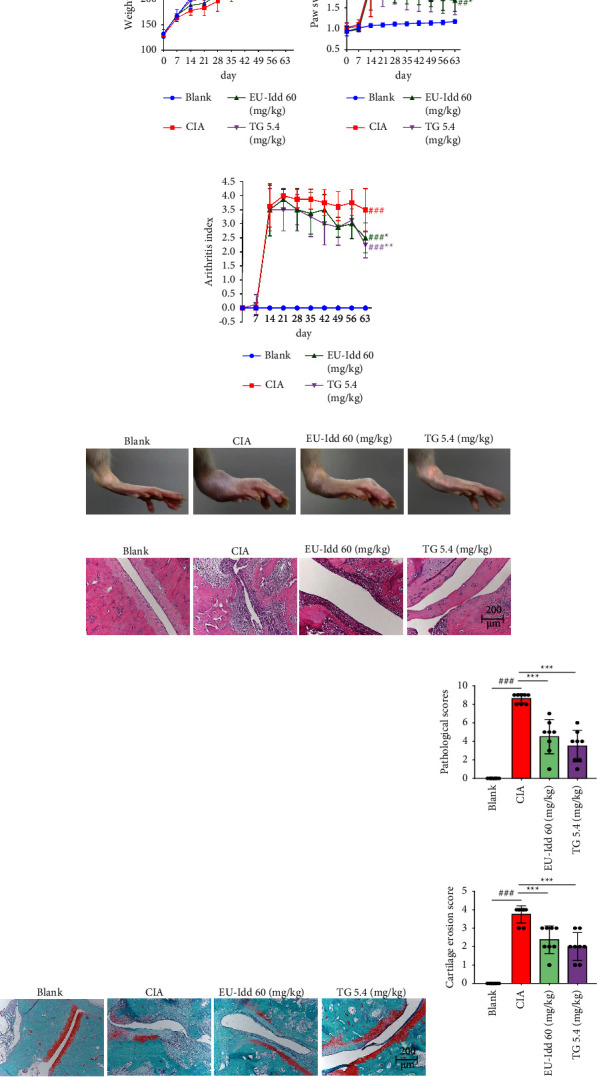
EU-Idd improved ankle joint pathology in CIA rats. (a) Changes in body weight of CIA rats (*n* = 8 for each group). The rats were weighed every 7 days. (b) Changes of paw swelling in CIA rats (*n* = 8 for each group). Paw volume was measured every 7 days and the mean value of the last two paw measurements was taken as the final paw volume. (c) Changes of arthritis score in CIA rats (*n* = 8 for each group). The arthritis index of each rat was recorded every 7 days on a scale of 0–4. (d) Representative images of paw swelling from each group at the time of the final administration (*n* = 8 for each group). (e) HE staining of pathological changes in the ankle joint tissue of CIA rats on day 63 (*n* = 8 for each group, magnification 200x). (f) Representative images of Safranin O-fast green staining of ankle tissue in rats after administration (*n* = 8 for each group, magnification 200x). (g) Histopathological scores and cartilage erosion scores of the ankle joints from each group after administration. Histopathological scores were scored on a scale of 0–4 based on pannus formation, synovial hyperplasia, and cartilage erosion. The cartilage erosion scores were graded on a scale of 0–4 according to the degree of cartilage damage. All data are expressed as mean ± SD. ^#^*P* < 0.05, ^##^*P* < 0.01, and ^###^*P* < 0.001*vs* Blank; ^*∗*^*P* < 0.05, ^*∗∗*^*P* < 0.01, and ^*∗∗∗*^*P* < 0.001*vs* CIA. Blank = blank control group. CIA = CIA control group. TG = *Tripterygium glycosides*.

We established a CIA rat model to evaluate the antiarthritic effects of EU-Idd. Changes in body weight of the rats in each group were measured every 7 days from the start of the experiment; the results are shown in Figure 2(a). There was no significant difference in body weight between the CIA group and each administration group. However, the fur color of rats in the CIA group was yellow and dull; these rats also ate less and were less active when compared with rats from the EU-Idd group. From the beginning of the experiment, the bilateral paw volume of the rats in each group was measured every 7 days. On the 14^th^ day of the experiment, the paw volume of the model group was significantly greater than that in the blank group (*P* < 0.05). As shown in Figure 2(b), the paw volume of the CIA group reached a peak on the 21^st^ day of the experiment. Following treatment with EU-Idd for 7 weeks, the paw volume was significantly downregulated. Moreover, the arthritis index decreased from the 21^st^ day (Figures 2(c) and 2(d)). Thus, iridoid exerts inhibitory effects on joint inflammation in CIA rats.

The results arising from HE staining are shown in Figure 2(e). The joint structure of rats in the blank group was clearly visible. However, we observed synovial tissue hyperplasia in rats from the CIA group and many inflammatory cells had infiltrated the ankle tissue. The administration of EU-Idd inhibited synovial hyperplasia of the ankle tissue and improved the pathology of the joint structure (Figure 2(g)). The articular cartilage in the ankle was stained by Safranin O-fast green staining; results are shown in Figure 2(f). After 7 weeks of EU-Idd treatment, the cartilage damage had reduced significantly; there was also a reduction in the cartilage score (Figure 2(g)).

#### 3.1.2. EU-Idd Relieved Bone Erosion in the Ankle Joints

**Figure 3:**
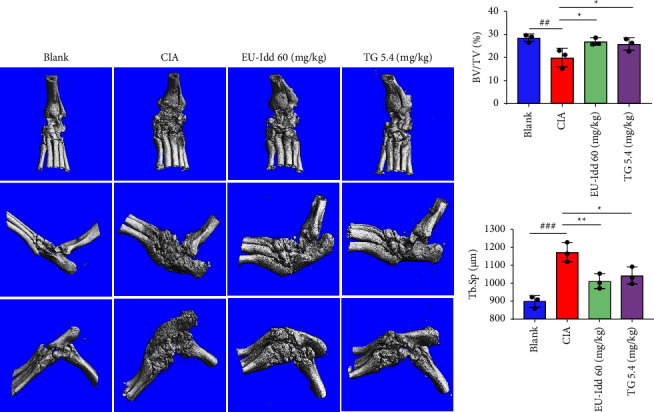
The effects of EU-Idd on bone erosion in CIA rats. (a) Representative reconstructed micro-CT images of the right hind paws of rats from the Blank, CIA, EU-Idd, and TG groups showing bone erosion (*n* = 3 for each group). The hind right paw of rats from the CIA group is shown in 3D. (b) Changes in trabecular bone volume (BV/TV) and trabecular separation (Tb.Sp) (*n* = 3 for each group). All data are expressed as mean ± SD. ^#^*P* < 0.05, ^##^*P* < 0.01, and ^###^*P* < 0.001*vs* Blank; ^*∗*^*P* < 0.05, ^*∗∗*^*P* < .01, and ^*∗∗∗*^*P* < 0.001*vs* CIA. Blank = blank control group. CIA = CIA control group. TG = *Tripterygium glycosides.*

Micro-CT scanning of the right hind paws of rats from each group provided high-resolution three-dimensional (3D) images and related bone parameters that were able to characterize the level of bone growth and development; results are shown in Figure 3(a). Compared with the CIA group, the EU-Idd treatment group showed less bone erosion, less joint damage, and a clearer joint structure. Bone parameters are shown in Figure 3(b). Compared with the Blank group, the ratio of bone volume per tissue volume (BV/TV) in the CIA group had decreased significantly (*P* < 0.01), thus proving that the bone mass had decreased. However, trabecular separation (Tb.Sp) had increased significantly (*P* < 0.001), thus proving that the mean width of the medullary cavity between the trabecular bone had increased. Conversely, the BV/TV values of the ankle joints from CIA rats increased after EU-Idd treatment while Tb.Sp values decreased.

#### 3.1.3. EU-Idd Reduced the Levels of Inflammation and Cytokines Related to Bone Metabolism in CIA Rats

**Figure 4:**
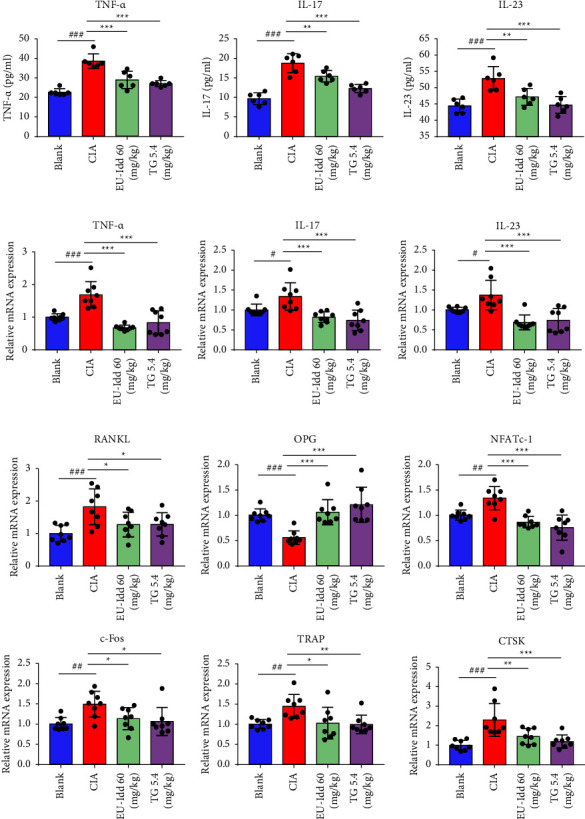
The effects of EU-Idd on the expression levels of TNF-*α*, IL-17, and IL-23 in serum and spleen along with bone metabolism-related factors in joint tissues from CIA rats. (a) The serum levels of TNF-*α*, IL-17, and IL-23 in rats from the Blank, CIA, EU-Idd, and TG groups were analyzed by ELISA (*n* = 6 for each group). (b) The mRNA expression levels of *TNF-α*, *IL-17*, and *IL-23* in the spleen of rats from the Blank, CIA, EU-Idd, and TG groups were detected by RT-qPCR (*n* = 8 for each group). (c) The mRNA expression levels of cytokines related to bone metabolism in joint tissues of rats from the Blank, CIA, EU-Idd, and TG group were detected by RT-qPCR (*n* = 6 for each group). All data are expressed as mean ± SD. ^#^*P* < 0.05, ^##^*P* < 0.01, and ^###^*P* < 0.001*vs* Blank; ^*∗*^*P* < 0.05, ^*∗∗*^*P* < 0.01, and ^*∗∗∗*^*P* < 0.001*vs* CIA. Blank = blank control group. CIA = CIA control group. TG = *Tripterygium glycosides.*

The serum concentrations of inflammatory cytokines (TNF-*α*, IL-17, and IL-23) were measured by ELISA; the results are shown in Figure 4(a). Compared with the blank group, the serum levels of TNF-*α*, IL-17, and IL-23 in the CIA group were significantly increased (*P* < 0.001). The trend for variation in inflammatory cytokines was consistent with the CIA inflammation model. The mRNA expression levels of *TNF-α*, *IL-17*, and *IL-23* in the spleen of rats from each group were detected by RT-qPCR (Figure 4(b)). We found that the trend for variation in mRNA expression for the three inflammatory cytokines in the spleen was similar to that in the serum. The administration of EU-Idd significantly downregulated the expression levels of TNF-*α*, IL-17, and IL-23 in the serum and the spleen (*P* < 0.01, *P* < 0.001). Collectively, these results suggested that EU-Idd had a certain anti-inflammatory effect.

To determine the role of EU-Idd on the expression of factors related to bone metabolism in the joint tissues of rats in each group, we next used RT-qPCR to detect the expression of key genes (Figure 4(c)). The mRNA expression levels of *RANKL*, nuclear factor of activated T cells cytoplasmic 1 (*NFATc-1)*, *c-Fos*, tartrate resistant acid phosphatase (*TRAP)*, and cathepsin K (*CTSK)* were significantly increased in the CIA rats. The expression levels of osteoprotegerin (*OPG)* mRNA, a gene related to osteoblastogenesis, were significantly decreased (*P* < 0.001). The administration of EU-Idd significantly downregulated the mRNA expression levels of *RANKL*, *NFATc-1*, *c-Fos TRAP*, and *CTSK* in articular cartilage (*P* < 0.05 or *P* < 0.01 or *P* < 0.001) and upregulated the mRNA expression levels of *OPG* (*P* < 0.001).

#### 3.1.4. EU-Idd Reduced the Proportions of Th17 Cells and the Phosphorylation of JAK2/STAT3 Proteins

**Figure 5:**
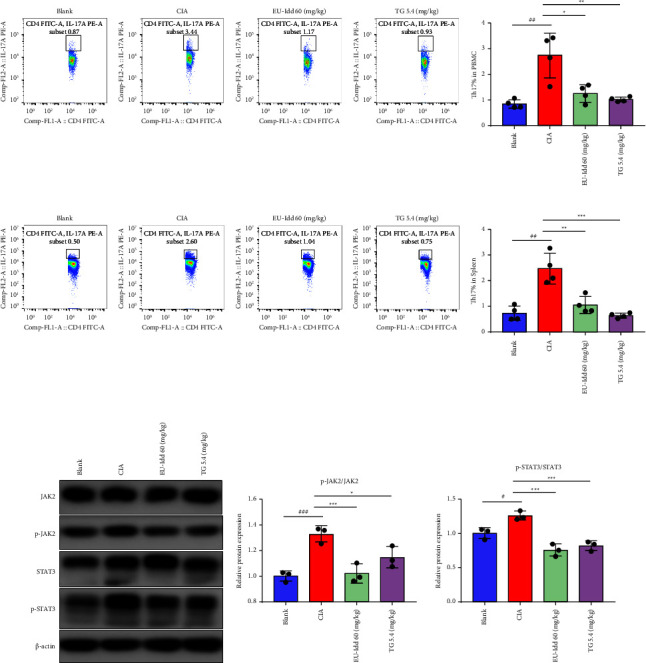
EU-Idd inhibited the proportions of Th17 cells and the phosphorylation of proteins in the JAK2/STAT3 pathway in the spleens of CIA rats. (a) The proportion of Th17 cells in the peripheral blood of rats in each group. (b) The proportion of Th17 cells in the spleens of rats in each group. (c) The protein expression levels of JAK2, p-JAK2, STAT3, and p-STAT3 in the spleens of rats in the Blank, CIA, EU-Idd, and TG groups were detected by western blotting (*n* = 8 for each group). (d) Densitometry scans of the levels of phosphorylation for JAK2 and STAT3 were analyzed and normalized to the total JAK2 and STAT3 proteins (*n* = 8 for each group). All data are expressed as mean ± SD. ^#^*P* < 0.05, ^##^*P* < 0.01, and ^###^*P* < 0.001*vs* Blank; ^*∗*^*P* < 0.05, ^*∗∗*^*P* < 0.01, and ^*∗∗∗*^*P* < 0.001*vs* CIA. Blank = blank control group. CIA = CIA control group. TG = *Tripterygium glycosides.*

The proportions of Th17 cells in the peripheral blood and spleens of rats in each group are shown in Figures 5(a) and 5(b). The proportions of Th17 cells in the peripheral blood and spleens of rats in the CIA group were significantly higher than those in the blank group (*P* < 0.01). The proportion of Th17 cells in the EU-Idd treatment group was significantly lower than that in the CIA group (*P* < 0.05, *P* < 0.01). Collectively, results suggested that EU-Idd inhibited the expression of Th17 cells and might play an important role in inhibiting joint inflammation in RA by regulating the production of Th17 cells. The protein expression levels of JAK2, p-JAK2, STAT3, and p-STAT3 in the spleens of rats from each group were detected by western blotting (Figure 5(c)). Next, we investigated the effect of EU-Idd on the JAK2/STAT3 pathway, as shown in Figure 5(d). In the CIA model group, the protein expression levels of p-JAK2/JAK2 and p-STAT3/STAT3 were significantly higher than those in the blank group (*P* < 0.05, *P* < 0.001). Conversely, compared with the CIA group, the administration of EU-Idd significantly downregulated the expression levels of p-JAK2 and p-STAT3 (*P* < 0.05, *P* < 0.01, *P* < 0.001), thus suggesting that EU-Idd effectively inhibited the phosphorylation and activation of the JAK2/STAT3 pathway.

### 3.2. EU-Idd Inhibited the Differentiation of CD4^+^ T Cells into Th17 Cells

#### 3.2.1. EU-Idd Reduced the Proportion of Th17 Cells in CD4^+^ T Cells

**Figure 6:**
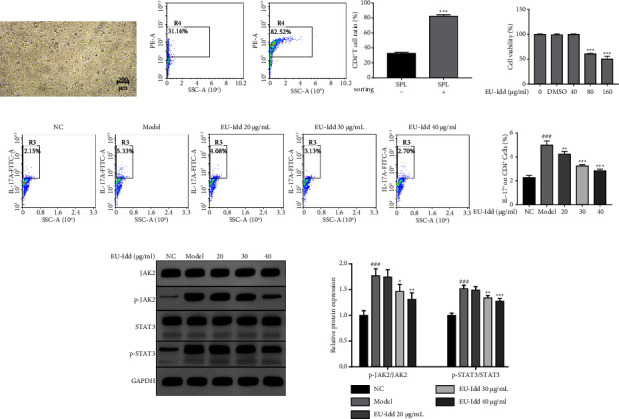
EU-Idd inhibited the differentiation of CD4^+^ T cells towards Th17 cells. (a) The morphology of CD4^+^ T cells. After magnetic bead sorting, the morphology of CD4^+^ T cells was observed under an inverted microscope and photographed, scale bar = 200 *μ*m. (b) The proportion of CD4^+^ T cells after magnetic bead sorting. The CD4^+^ T cells were enriched by magnetic bead sorting and the proportion of CD4^+^ T cells after sorting was detected by flow cytometry. The data are expressed as mean ± SD. ^*∗*^*P* < 0.05, ^*∗∗*^*P* < 0.01, and ^*∗∗∗*^*P* < 0.001*vs* unsorted CD4^+^ T cells. (c) The effect of different concentrations of EU-Idd (20, 30, and 40 *μ*g/mL) on the viability of CD4^+^ T cells within 24 h. The CD4+T cells used for the CCK-8 assay were isolated from normal rats to investigate the effect on cell viability caused by modeling. The experiment was repeated three times. The data are expressed as mean ± SD (*n* = 4 for each group). ^*∗*^*P* < 0.05, ^*∗∗*^*P* < 0.01, and ^*∗∗∗*^*P* < 0.001*vs* NC. (d) Effects of different concentrations of EU-Idd (20, 30, and 40 *μ*g/mL) on Th17 differentiation. The CD4^+^ T cells of CIA rats were induced to differentiate into Th17 cells with stimulatory factors *in vitro.* After 5 days of induction, the proportion of Th17 cells in each group was detected by flow cytometry. (e) The effects of EU-Idd on the protein expression of JAK2, p-JAK2, STAT3, and p-STAT3 during Th17 cell differentiation. The data are expressed as mean ± SD (*n* = 3 for each group). ^#^*P* < 0.05, ^##^*P* < 0.01, and ^###^*P* < 0.001*vs* NC; ^*∗*^*P* < 0.05, ^*∗∗*^*P* < 0.01, and ^*∗∗∗*^*P* < 0.001*vs* model. NC, the non-induced group. Model = the induced group. The CD4^+^ T cells used for assays were isolated from CIA rats.

To obtain more CD4^+^ T cells, we performed magnetic bead sorting experiments; results are shown in Figures 6(a) and 6(b). The morphology of CD4^+^ T cells was observed under an inverted microscope; cell morphology appeared to be relatively uniform, round, and granular, and the cells did not adhere to the wall. After sorting, the cell purity was greater than 80%. After 40, 80, and 160 *μ*g/mL of EU-Idd was applied to the CD4^+^ T cells for 24 h, we used the CCK-8 kit to detect the effect of EU-Idd on the viability of CD4^+^ T cells; results are shown in Figure 6(c). Compared with the NC group, there was no significant difference in the survival rate of cells in the DMSO group (*P* > 0.05). At concentrations of 80 and 160 *μ*g/mL, EU-Idd significantly reduced the viability of CD4^+^ T cells. When the concentration was around 40 *μ*g/mL, there was no significant difference in cell viability when compared with the NC group (*P* > 0.05). Therefore, 20, 30, and 40 *μ*g/mL of EU-Idd were selected for subsequent experiments. When induced by specific stimulatory factors and antibodies, CD4^+^ T cells were directed to differentiate into Th17 cells; the proportion of Th17 cells after induction was identified by flow cytometry, as shown in Figure 6(d). Compared with the NC group, the proportion of Th17 cells in the model group was significantly increased (*P* < 0.001). In the EU-Idd administration group, there was a significant reduction in the number of Th17 cells; this occurred in a concentration-dependent manner (20, 30, and 40 *μ*g/mL). These results suggested that EU-Idd negatively regulated the differentiation of Th17 cells.

#### 3.2.2. EU-Idd Inhibited the Phosphorylation of the JAK2/STAT3 Signaling Pathway during the Differentiation of Th17 Cells

To investigate the mechanism by which EU-Idd inhibited the differentiation of Th17 cells *in vitro*, we used western blotting to detect the phosphorylated protein expression levels of JAK2 and STAT3 in Th17 cells after directional induction for 5 days. The results are shown in Figure 6(e). Compared with the NC group, the model group showed significantly increased protein expression levels of p-JAK2 and p-STAT3 (*P* < 0.001). Compared with the model group, the 20 *μ*g/mL EU-Idd group showed downregulated expression levels of both JAK2 and STAT3 phosphorylated proteins, although these differences were not statistically significant (*P* > 0.05). Compared with the model group, the EU-Idd (30, 40 *μ*g/mL) group showed significant reductions in the protein expression levels of p-JAK2 and p-STAT3 (*P* < 0.05 or *P* < 0.01 or *P* < 0.001).

### 3.3. Effects of EU-Idd on Osteoclast Differentiation

#### 3.3.1. EU-Idd Effectively Inhibited the Differentiation of RAW264.7 Cells into Osteoclasts

**Figure 7:**
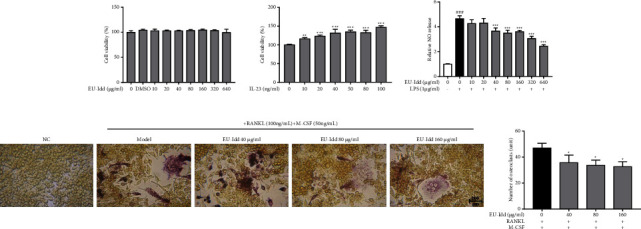
The effects of EU-Idd on NO release and osteoclast differentiation in RAW264.7 cells. (a) The cell viability of RAW264.7 cells treated with EU-Idd (0, 10, 20, 40, 80, 160, 320, and 640 *μ*g/mL) for 24 h. (b) The cell viability of RAW264.7 cells treated with IL-23 (0, 10, 20, 40, 50, 80, and 100 *μ*g/mL) for 24 h. (c) The amount of NO released in the supernatant of cells stimulated by LPS after EU-Idd intervention for 24 h. All data are expressed as mean ± SD (*n* = 4 for each group). ^#^*P* < 0.05, ^##^*P* < 0.01, and ^###^*P* < 0.001*vs* NC; ^*∗*^*P* < 0.05, ^*∗∗*^*P* < 0.01, and ^*∗∗∗*^*P* < 0.001*vs* LPS. NC, the non-induced group. LPS = the induced group. (d) Osteoclast TRAP staining. RAW264.7 cells were stimulated with RANKL (100 ng/mL) and M-CSF (50 ng/mL) for 7 days; then, the cells were stimulated by IL-23 (20 ng/mL) and EU-Idd (40, 80, and 160 *μ*g/mL) for 24 h. Following stimulation, multi-nucleated osteoclasts were selected under the microscope for counting and imaging, scale bar = 200 *μ*m. All data are expressed as mean ± SD (*n* = 4 for each group). ^*∗*^*P* < 0.05, ^*∗∗*^*P* < 0.01, and ^*∗∗∗*^*P* < 0.001*vs* model. Model, the induced group.

RAW264.7 cells were treated with EU-Idd at concentrations ranging from 10 to 640 *μ*g/mL and IL-23 at concentrations ranging from 0 to 100 ng/ml; cell viability was then detected after 24 h, as shown in Figures 7(a) and 7(b). EU-Idd showed no significant effect on cell viability (*P* > 0.05) while IL-23 significantly promoted the proliferation of RAW264.7 cells when the concentration was greater than 10 ng/ml (*P* < 0.01 or *P* < 0.001). RAW264.7 cells were further stimulated with LPS; we found that at concentrations ranging from 40 to 640 *μ*g/mL, EU-Idd effectively reduced the amount of NO released in the supernatant of cells after stimulation (Figure 7(c)). Combined with the cell viability detection experiment, 40, 80, and 160 *μ*g/mL of EU-Idd were used for subsequent experiments. After inducing RAW264.7 cells to differentiate into osteoclasts for 7 days, the effects of EU-Idd at concentrations of 40, 80, and 160 *μ*g/mL on osteoclast differentiation were observed by TRAP staining; the experimental results are shown in Figure 7(d). Cells in the NC group were not confluent and exhibited a monocyte state. The nuclei of the induced RAW264.7 cells were fused and the osteoclasts were stained purple/red. Most of the osteoclasts exhibited 3–8 nuclei. Compared with the model group that was induced to differentiate but without EU-Idd, the different concentrations of EU-Idd significantly reduced the number of osteoclasts (*P* < 0.05).

#### 3.3.2. EU-Idd Inhibited JAK2/STAT3 Phosphorylation Activation in Osteoclasts and Regulated the Expression Levels of Proteins Related to Bone Metabolism

**Figure 8:**
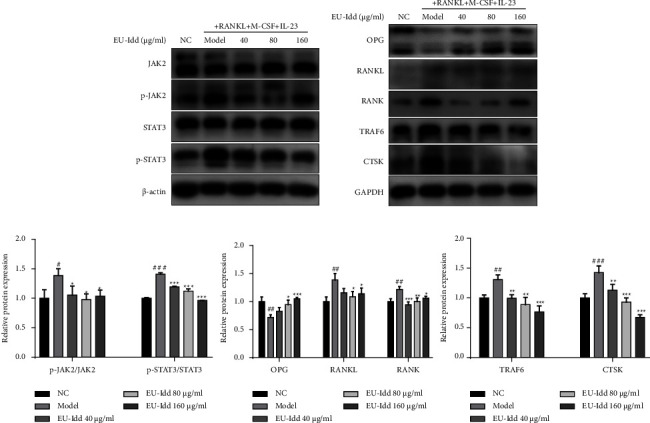
The effects of EU-Idd on the expression levels of proteins in the JAK2/STAT3 pathway and pathways related to bone metabolism. (a) Protein expression levels of JAK2, p-JAK2, STAT3, and p-STAT3 after treatment with different concentrations of EU-Idd during the differentiation of osteoclast cells, as measured by western blotting. (b) The protein levels of OPG, RANKL, RANK, TRAF6, and CTSK were determined by western blotting. (c) Densitometry was performed to determine the levels of JAK2 and STAT3 phosphorylation and data were normalized to the total protein levels of JAK2 and STAT3 (*n* = 4 for each group). Densitometry data for OPG, RANKL, RANK, TRAF6, and CTSK were normalized to GAPDH, respectively (*n* = 4 for each group). All data are expressed as mean ± SD. ^#^*P* < 0.05, ^##^*P* < 0.01, and ^###^*P* < 0.001*vs* NC; ^*∗*^*P* < 0.05, ^*∗∗*^*P* < 0.01, and ^*∗∗∗*^*P* < 0.001*vs* model. NC, the non-induced group. Model, the induced group.

The effects of EU-Idd (40, 80, 160 *μ*g/mL) on the expression levels of protein in the JAK2/STAT3 pathway and proteins related to bone metabolism during the differentiation of RAW264.7 cells into osteoclasts were observed by western blotting; results are shown in Figures 8(a) and 8(b). After 7 days of induction, compared with the NC group, the model group induced by RANKL, M-CSF, and IL-23 but without EU-Idd showed a significant increase in the protein expression levels of p-JAK2 and p-STAT3 (*P* < 0.05, *P* < 0.001); EU-Idd (40, 80 and 160 *μ*g/mL) significantly inhibited the phosphorylation activation of the JAK2/STAT3 pathway in cells (*P* < 0.05, *P* < 0.001), as shown in Figure 8(c). The model group showed a significant increase in the protein expression levels of RANKL, RANK, TRAF6, and CTSK; these proteins are all related to osteoclastogenesis (*P* < 0.01, *P* < 0.001). There was also a significant reduction in the protein expression levels of OPG, a protein related to osteoblastogenesis (*P* < 0.01, *P* < 0.001). Conversely, different concentrations of EU-Idd significantly reduced the protein expression levels of RANKL, RANK, TRAF6, and CTSK and significantly increased the expression levels of OPG protein (*P* < 0.05, *P* < 0.001). Analysis showed that 40 *μ*g/mL of EU-Idd improved protein levels in the RANKL/OPG axis although these changes were not significantly different (*P* > 0.05).

### 3.4. The Inhibitory Effects of EU-Idd on Inflammation in HFLS-RA Cells

#### 3.4.1. EU-Idd Inhibited the Migration and Invasion of HFLS-RA Cells

**Figure 9:**
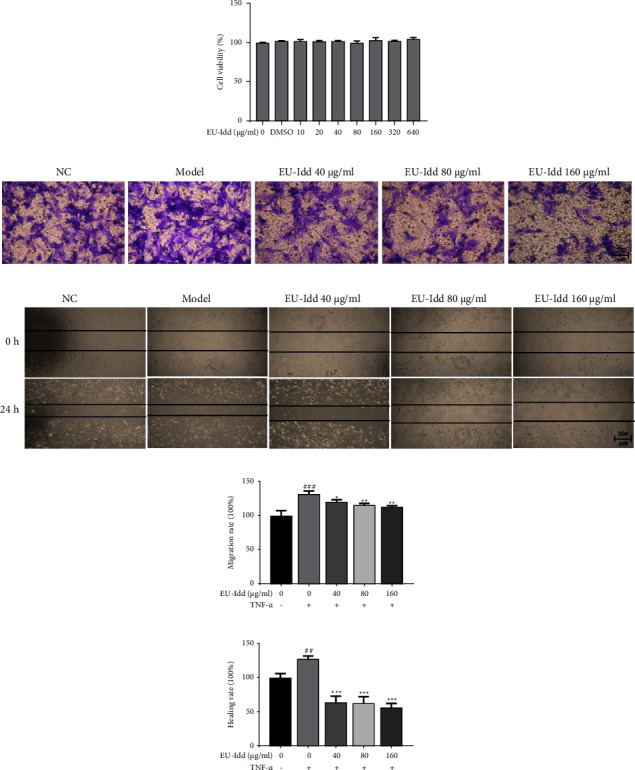
EU-Idd inhibited the invasion and migration of TNF-*α*-induced HFLS-RA cells. (a) The effects of EU-Idd (0, 10, 20, 40, 80, 160, 320, and 640 *μ*g/mL) on the viability of HFLS-RA cells for 24 h. (b) Representative photomicrographs of cell migration; cells were stimulated with TNF-*α* (20 ng/mL) for 24 h and treated with EU-Idd (40, 80, and 160 *μ*g/mL). Five fields of view from each group were used for imaging, scale bar = 200 *μ*m. (c) The effect of EU-Idd on the wound migration of HFLS-RA cells. The cells were photographed under an inverted microscope at 0 h and 24 h; we also recorded the scratched areas, scale bar = 200 *μ*m. (d) EU-Idd attenuated HFLS-RA migration rate at concentrations of 40, 80, and 160 *μ*g/mL. Cells were eluted with 33% acetic acid and measured at 595 nm (*n* = 3 for each group). (e) EU-Idd (40, 80, and 160 *μ*g/mL) inhibited the HFLS-RA healing rate (*n* = 3 for each group). All data are expressed as mean ± SD. ^#^*P* < 0.05, ^##^*P* < 0.01, and ^###^*P* < 0.001*vs* NC; ^*∗*^*P* < 0.05, ^*∗∗*^*P* < 0.01, and ^*∗∗∗*^*P* < 0.001*vs* model. NC, the non-induced group. Model, the induced group.

To explore the anti-inflammatory activity of EU-Idd*in vitro*, we used TNF-*α* to stimulate HFLS-RA cells and observed the effect of EU-Idd administration. Cell viability assays showed that EU-Idd had no significant effect on the viability of HFLS-RA cells at concentrations ranging from 10 to 640 *μ*g/mL (*P* > 0.05) (Figure 9(a)). Since EU-Idd (at concentrations of 40, 80, and 160 *μ*g/mL) inhibited the NO content in the supernatant of RAW264.7 cells and the differentiation of osteoclasts, we chose concentrations of 40, 80, and 160 *μ*g/mL to investigate anti-inflammatory effects on HFLS-RA cells. The effects of different concentrations of EU-Idd administration on the invasion of HFLS-RA cells were determined by Transwell assays; results are shown in Figure 9(b). After staining, a greater number of stained cells were observed in the model group; in addition, the invasion rate was significantly higher than that of the NC group (*P* < 0.001) (Figure 9(d)). EU-Idd (40, 80, and 160 *μ*g/mL) reduced the rate of invasion and inhibited the invasion of HFLS-RA cells after TNF-*α* induction. The effect of EU-Idd group on the migration ability of HFLS-RA cells was judged by scratch assays; the results are shown in Figure 9(c). At 0 h, the size of the scratched area in each group was similar. After 24 h of TNF-*α* stimulation, the scratched area in the model group was significantly smaller (*P* < 0.01) (Figure 9(e)); the scratched area was significantly increased while the scratch healing rate was significantly slower (*P* < 0.001).

#### 3.4.2. EU-Idd Downregulated the Levels of Related Inflammatory Cytokines in HFLS-RA Cells

**Figure 10:**
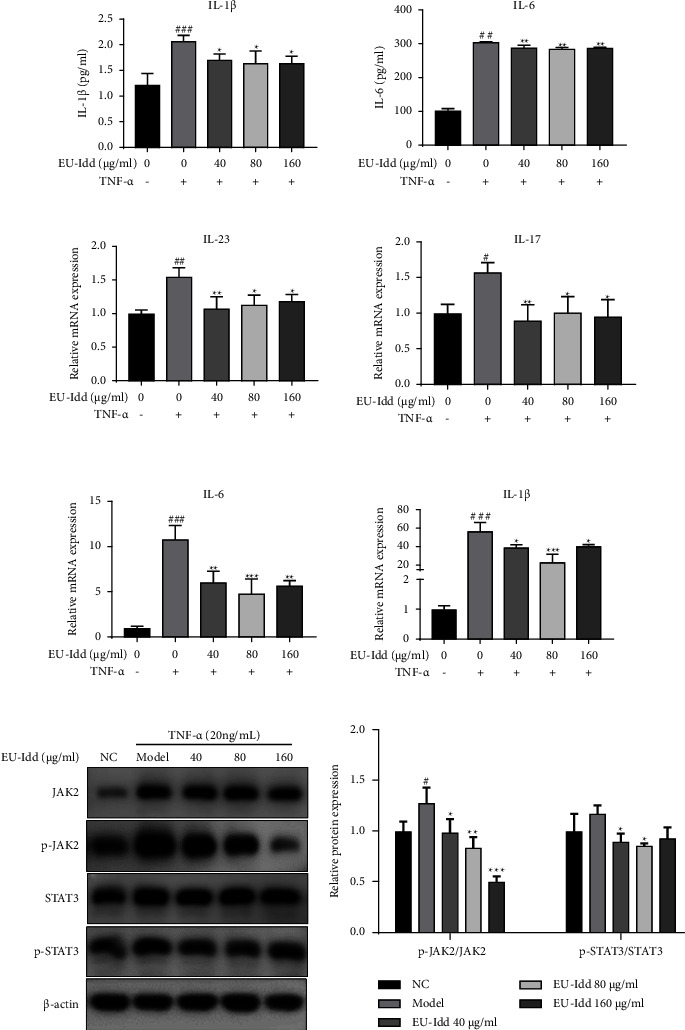
EU-Idd inhibited the expression of inflammatory cytokines, p-JAK2, and p-STAT3 in TNF-*α*-induced HFLS-RA cells. (a) The effects of EU-Idd (40, 80, and 160 *μ*g/mL) on the expression levels of IL-1*β* and IL-6 in cell supernatants were detected by ELISA. (b) EU-Idd inhibited the mRNA expression levels of *IL-23*, *IL-17*, *IL-6*, and *IL-1β* in cells; the mRNA levels of cytokines were normalized to *β*-actin. (c) Western blotting was used to detect JAK2, p-JAK2, STAT3, and p-STAT3 protein expression levels in HFLS-RA cells. (d) Densitometry scans for the phosphorylated levels of JAK2 and STAT3 were analyzed and normalized to the total proteins of JAK2 and STAT3 (*n* = 4 for each group). All data are expressed as mean ± SD. ^#^*P* < 0.05, ^##^*P* < 0.01, and ^###^*P* < 0.001 vs NC; ^*∗*^*P* < 0.05, ^*∗∗*^*P* < 0.01, and ^*∗∗∗*^*P* < 0.001*vs* model. NC, the non-induced group. Model, the induced group.

The effects of EU-Idd on the expression of inflammatory cytokines in HFLS-RA cells were detected by ELISA and RT-qPCR. As shown in Figure 10(a), the levels of IL-1*β* and IL-6 in the supernatant of cells stimulated by TNF-*α* for 24 h were significantly increased (*P* < 0.01 or *P* < 0.001). EU-Idd inhibited the release of IL-1*β* and IL-6 from cells (*P* < 0.05 or *P* < 0.01). As shown in Figure 10(b), the mRNA expression of *IL-17*, *IL-6*, *IL-23,* and *IL-1β* in cells was significantly increased after TNF-*α* induction, especially *IL-6* and *IL-1β*. However, at concentrations of 40, 80, and 160 *μ*g/mL, EU-Idd significantly inhibited the mRNA expression levels of *IL-17*, *IL-6*, *IL-23*, and *IL-1β* (*P* < 0.05, *P* < 0.01, *P* < 0.001).

#### 3.4.3. EU-Idd Inhibited the Phosphorylation of JAK2 and STAT3 in HFLS-RA Cells

Total protein was extracted from HFLS-RA cells for western blot assays to explore the mechanisms underlying the anti-inflammatory effects of EU-Idd; results are shown in Figure 10(c). Compared with the NC group, the expression levels of p-JAK2 protein were significantly increased after stimulation with TNF-*α* (*P* < 0.05). The expression levels of p-STAT3 protein were increased but with no statistical significance (*P* > 0.05). EU-Idd (40, 80, and 160) significantly reduced the phosphorylated protein expression level of JAK2 (*P* < 0.05, *P* < 0.01, and *P* < 0.001). EU-Idd significantly reduced the expression levels of p-STAT3 protein at concentrations of 40 and 80 *μ*g/mL, although the downregulation effect of EU-Idd at 160 *μ*g/mL was not significant (*P* > 0.05).

## 4. Discussion

The iridoid component of *E. ulmoides* has been shown to exhibit positive anti-inflammatory activity and is mainly composed of geniposidic acid, geniposide, and aucubin [15–18]. Geniposide has been demonstrated to reduce inflammation and immunological control, enhance mitochondrial apoptosis in MC3T3-E1 cells, and have bone-protecting property [19, 20]. According to the arthritis research of aucubin, it plays an important role in reducing cartilage destruction and preventing apoptosis in chondrocytes in a mouse model of OA, and aucubin has also been proved to exert therapeutic effects on CIA rats and accelerate synovial cell death [21, 22].

In CIA model rats, we discovered that EU-Idd had an intervention effect on inflammation. Additionally, EU-Idd decreased the paw volume and arthritis index in rats, improved the pathology of the ankle joints, and had a bone-protective effect. Moreover, it has been shown that EU-Idd negatively regulates the level of expression of associated inflammatory cytokines and proteins involved in bone metabolism.

Prior to bone destruction, the principal pathological manifestation is that the synovial tissue is infiltrated by many inflammatory cells; there is also the secretion of pro-inflammatory cytokines. Naive CD4^+^ T cells can differentiate into Treg and Th1, Th2, and Th17 cells under different stimulation conditions. Imbalances in the ratio and function of various Th cell subsets and between Th17 cells and Treg cells play a crucial role in the pathogenesis of RA [23]. Th17 is known to produce a marked effect in RA by producing IL-17, a cytokine that can stimulate synovial fibroblasts to produce a variety of inflammatory factors, thus resulting in the induction of synovitis [24]. Moreover, IL-17 activates the expression of RANKL, a protein related to osteoclastogenesis in monocytes, and disrupts bone homeostasis [6]. In this study, we noticed that the ratio of Th17 cells in the peripheral blood and spleens of rats in the CIA model was significantly higher than that of rats in the blank group. To further verify the effect of EU-Idd on Th17 differentiation *in vitro*, we directed the differentiation of CD4^+^ T cells from CIA rats to Th17 cells. TGF-*β* activates downstream STAT3 together with IL-6; in turn, this leads to high expression levels of ROR*γ*t, which initiates the differentiation cascade of Th17 cell [25]. According to experimental methods described in the existing literature [26, 27], we used cytokine stimulation (TGF-*β*, IL-6, TNF-*α*, IL-1*β*, and IL-23) under coating conditions created by CD3 and CD28 antibodies to induce Th17 cells. EU-Idd treatment significantly inhibited the differentiation of Th17 cells. Based on these results, it appeared that EU-Idd reduced the production of Th17 cells and could regulate the immune system. To better explore the anti-inflammatory activity of EU-Idd, we detected changes in related inflammatory cytokines. IL-23 is a heterodimeric cytokine composed of *p*19, IL-12, and IL-23p40 connected by disulfide bonds. Studies have found that IL-23 is a cytokine that maintains Th17 expansion and stability [28]. IL-23 exerts its biological effects mainly by binding to its receptor IL-23R to stimulate cells to produce inflammatory factors and activate related signaling pathways [29]. A significant cytokine in the inflammatory response of RA is TNF-*α*. TNF-*α* mainly causes cartilage damage in RA by promoting the release of various inflammatory mediators, the degradation of cartilage proteoglycan, and the expression of vascular endothelial cell adhesion molecules [30]. In rheumatoid arthritis, the chronic inflammation of synovial cells is largely caused by IL-1*β* overproduction; this directly activates FLS and leads to joint destruction and bone resorption [31, 32]. One of the most significant pro-inflammatory factors in the course of many inflammatory reactions is IL-6. Furthermore, IL-6 promotes the recruitment of inflammatory cells such as neutrophils into the synovium and promotes the expression of RANKL in synovial cells to stimulate the generation of osteoclasts [33]. We discovered that EU-Idd prevented the infiltration of inflammatory cells in the ankle joints and the expression of IL-23 and TNF-*α* of CIA rats and decreased the invasion and migration of HFLS-RA cells. The protein levels of IL-6 and IL-1*β* in HFLS-RA and the mRNA expression levels of *IL-6*, *IL-1β*, *IL-17*, and *IL-23* in HFLS-RA were downregulated by EU-Idd.

The inflammatory reaction was followed by bone destruction. In the early stages of RA, the symptoms of inflammation are obvious. We discovered that the paw volume of CIA rats had dramatically risen. The paw volume stabilized after 42 days. Micro-CT scanning further showed that the bone destruction in CIA rats was severe. Levels of cytokines associated with osteoclasts had also increased significantly. RANKL is involved in bone metabolism in regulating the differentiation of osteoclasts. RANKL acts directly on osteoclast precursors *via* its receptor RAN which binds to its receptor RANK and recruits TNF-receptor-associated factor 6 (TRAF6) to activate NF-*κ*B, NFATc-1, c-Fos, CTSK, and other major regulators related to osteoclast differentiation, to induce the differentiation of precursors into osteoclasts [34, 35]. OPG is a soluble decoy receptor of RANKL that can bind to RANKL to inhibit the activation of RANK and has an important role in protecting the bones; it can also negatively regulate the production of osteoclasts [36]. Both c-Fos and NFATc-1 are key transcription factors for the differentiation of osteoclasts and can initiate the expression of TRAP; CTSK can reflect the resorption activity of osteoclasts [37, 38]. *In vivo*, EU-Idd effectively increased the expression of *OPG* mRNA in CIA rats and reduced the mRNA expression of osteoclast-related cytokines such as *RANKL*. *In vitro*, EU-Idd effectively inhibited the differentiation of RAW264.7 cells into osteoclasts, reduced the protein expression of RANKL, RANK, TRAF6, and CTSK, and increased the protein expression of OPG. Collectively, these data suggested that EU-Idd inhibited the formation of osteoclasts and exerted a protective effect in bone.

Recent studies have shown that the JAK2/STAT3 pathway is a key factor in the inflammatory response and bone destruction of RA. Research targeting this pathway could lead to the development of more effective treatments for RA [39]. JAK2 is activated by autophosphorylation; following activation, it can form multiple receptor binding sites and present these sites for STAT3 for phosphorylation and dissociation into the nucleus to regulate the expression of genes related to the transcription factor ROR*γ*t, and it is essential for the differentiation of Th17 cells [40, 41]. Following the activation of the JAK2/STAT3 pathway, memory T lymphocytes can be induced to differentiate into Th17 cells to play a role in maintaining and expanding Th17 cells. Research has shown that Th17 cells produce a variety of pro-inflammatory mediators, including IL-17, IL-6, and IFN-*γ*, and IL-6 can further strengthen STAT3 signaling, promote the further differentiation of Th17 cells, and lead to the continuous deterioration of the inflammatory response [42]. In our study, phosphorylation of the JAK2/STAT3 protein was shown to be inhibited in CIA rats, while JAK2 and STAT3 were phosphorylated during CD4^+^ T cell differentiation into Th17 cells. EU-Idd suppresses the protein expression levels of p-JAK2 and p-STAT3 and cellular differentiation into Th17. EU-Idd also inhibited the phosphorylation and activation of the JAK2/STAT3 pathway in HFLS-RA cells stimulated by TNF-*α*. In terms of bone metabolism, previous researchers found that the protein expression levels of JAK2 and STAT3 were correlated with RANKL levels during osteoclastogenesis. Additionally, inhibiting the JAK2/STAT3 pathway and fostering Treg-mediated bone immunity can suppress osteoclast differentiation and bone resorption [43–46]. In this study, we observed that EU-Idd reduced the expression levels of p-JAK2 and p-STAT3 during the differentiation of RAW264.7 cells into osteoclasts induced by RANKL. However, some limitations should be noted. One limitation is that we have not yet explored the optimal therapeutic dose of EU-Idd for rheumatoid arthritis, and another limitation is that our results have not been verified by using JAK2/STAT3 pathway inhibitor. With the in-depth study of *E. ulmoides* and rheumatoid arthritis, it could lead to new targets aimed at treatment of rheumatoid arthritis.

## 5. Conclusion

EU-Idd could significantly inhibit joint inflammation and reduce the expression of inflammatory cytokines such as IL-17 and IL-23 and relieve bone erosion in the ankle joints in CIA rats. *In vitro*, EU-Idd inhibited the differentiation of CD4+ T cells into Th17 cells and the differentiation of RAW264.7 cells into osteoclasts and suppressed the migration and invasion of HFLS-RA cells. Collectively, our results indicate that EU-Idd inhibited the inflammatory response of CIA rats and reduced the production of osteoclasts *via* the JAK2/STAT3 pathway. Consequently, EU-Idd has significant potential for the treatment of rheumatoid arthritis.

## Figures and Tables

**Table 1:** The primer sequences used for RT-qPCR.

Gene	Sequence(5′-3′)	
Rat	GAPDH	F: CCACCCATGGCAAATTCCATGGCA
		R: TCTAGACGGCAGGTCAGGTCCACC
	TNF-*α*	F: GGAAAGCATGATCCGAGATG
		R: CGAGCAGGAATGAGAAGAGG
	IL-23	F: CAGTGTGGTGATGGTTGTGATCCC
		R: AGATGTCCGAGTCCAGCAGGTG
	IL-17	F: GCCGAGGCCAATAACTTTCT
		R: AGCCACAAATCTCAGGGTGG
	OPG	F: GGCAGGGCATACTTCC TGTT
		R: GCCACTTGTTCATTGTGGTCC
	RANKL	F: AGGCTGGGCCAAGATCTCTA
		R: GATAGTCCGCAGGTACGCTC
	TRAP	F: CCATTGTTAGCCACATACGG
		R: CACTCAGCACATAGCCCACA
	NFATc-1	F: GGAGAGTCCGAGAATCGAGAT
		R: TTGCAGCTAGGAAGTACGTCT
	c-FOS	F: TTTCAACGCCGACTACGAGG
		R: GCGCAAAAGTCCTGTGTGTT
	CTSK	F: TAGCACCCTTAGTCTTCCGC
		R: CTTGAACACCCACATCCTGC

Human	GAPDH	F: CATGAGAAGTATGACAACAGCCT
		R: AGTCCTTCCACGATACCAAAGT
	IL-23	F: CTCAGGGACAACAGTCAGTTC
		R: ACAGGGCTATCAGGGAGCA
	IL-17	F: TCCCACGAAATCCAGGATGC
		R: GGATGTTCAGGTTGACCATCAC
	IL-1*β*	F: TTCGACACATGGGATAACGAGG
		R: TTTTTGCTGTGAGTCCCGGAG
	IL-6	F: ACTCACCTCTTCAGAACGAATTG
		R: CCATCTTTGGAAGGTTCAGGTTG

## Data Availability

The data used to support the findings of this study are available from the corresponding author upon request.

## Supplementary Materials

Supplementary Materials Figure S1: (a) HPLC chromatograms of iridoids of *Eucommia ulmoides* (AU, aucubin; GA, geniposidic acid; GE, geniposide); (b) the content analysis of iridoids of *Eucommia ulmoides* by HPLC. Figure S2: changes of paw swelling in CIA rats (*n* = 8 for each group).
