# Supplementary material for: Iridoid from Eucommia ulmoides Oliv. Exerts Antiarthritis Effects by Inhibiting the JAK2/STAT3 Signaling Pathway In Vivo and In Vitro
Source: Evid Based Complement Alternat Med. 2023 Apr 19;2023:4167906. doi: 10.1155/2023/4167906 (PMC10132903; doi:10.1155/2023/4167906)
Supplement: Supplementary Materials — Figure S1: (a) HPLC chromatograms of iridoids of Eucommia ulmoides (AU, aucubin; GA, geniposidic acid; GE, geniposide); (b) the content analysis of iridoids of Eucommia ulmoides by HPLC. Figure S2: changes of paw swelling in CIA rats (n = 8 for each group). [file 4167906.f1.docx]

The Supplementary Material for this article is given in Fig. S1 and Fig. S2.


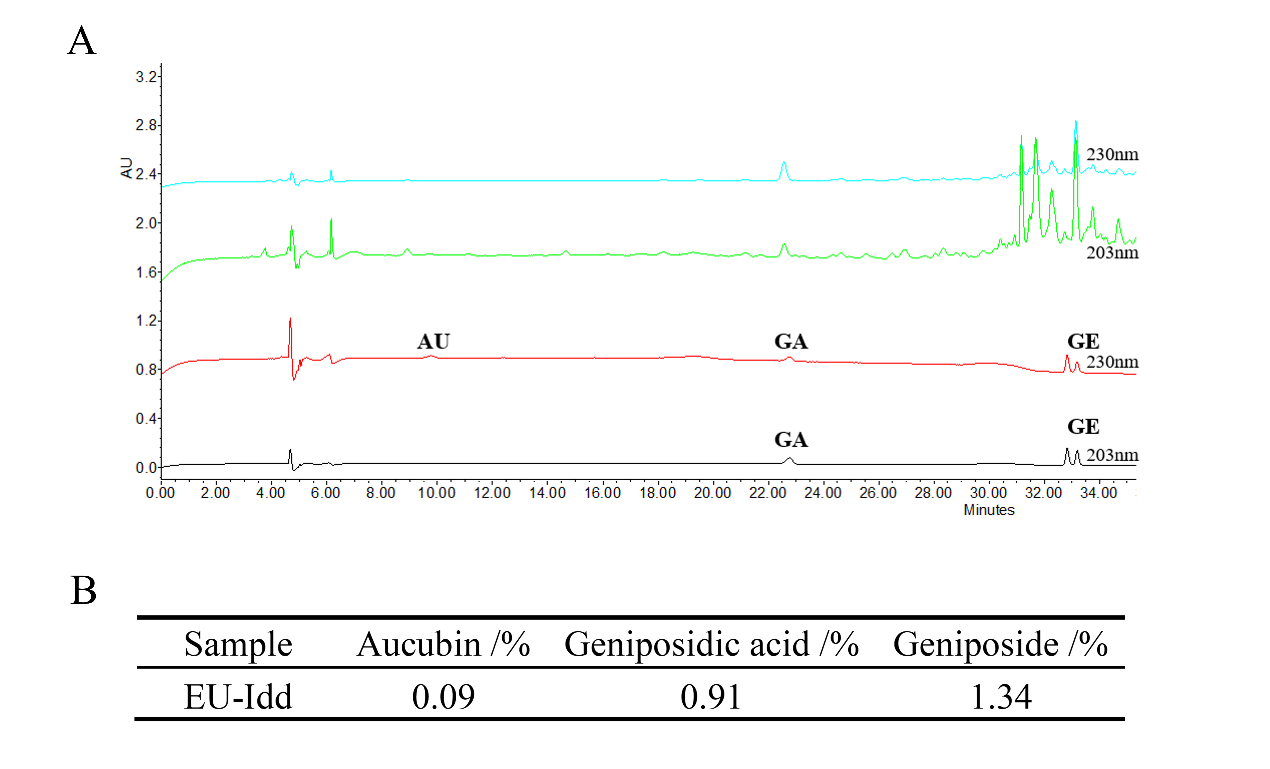


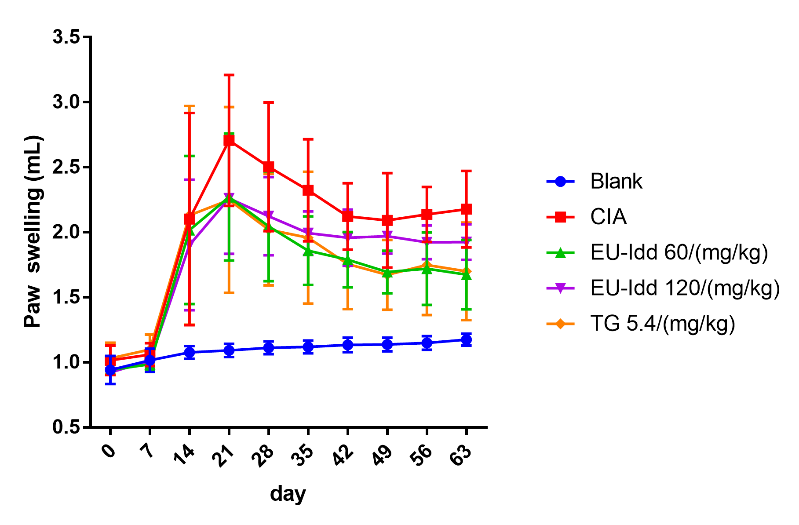
**Fig. S1 A** HPLC chromatograms of iridoids of *Eucommia ulmoides.*Aucubin(AU), Geniposidic acid(GA), Geniposide(GE).**B** The content analysis of iridoids of *Eucommia ulmoides* by HPLC*.*

**Fig. S2** Changes of paw swelling in CIA rats (n=8 for each group)
